# Gelatin-Based Microspheres for Sustained Ketoprofen Delivery in Difficult-to-Heal Wounds

**DOI:** 10.3390/polym18151807

**Published:** 2026-07-23

**Authors:** Chiara Kodra, Alessia Nito, Emma Quarta, Morena Miciaccia, Maria Grazia Perrone, Antonio Scilimati, Alessandro Sannino, Luca Salvatore, Nunzia Gallo

**Affiliations:** 1 Department of Engineering for Innovation, University of Salento, Via Monteroni, 73100 Lecce, Italy; c.kodra@typeone.it; 2 Typeone Biomaterials S.r.l., Via Europa 167, 73021 Calimera, Italy; l.salvatore@typeone.it; 3 CNR NANOTEC-Institute of Nanotechnology, c/o Campus Ecotekne, University of Salento, Via Monteroni, 73100 Lecce, Italy; alessia.nito@unisalento.it (A.N.); emmaquarta@cnr.it (E.Q.); 4 Medicinal Chemistry Research Laboratory for Woman and Child Health, Department of Pharmacy-Pharmaceutical Sciences, University of Bari Aldo Moro, Via E. Orabona 4, 70125 Bari, Italy; morena.miciaccia@uniba.it (M.M.); mariagrazia.perrone@uniba.it (M.G.P.); antonio.scilimati@uniba.it (A.S.); 5 Department of Experimental Medicine, University of Salento, Via Monteroni, 73100 Lecce, Italy; alessandro.sannino@unisalento.it

**Keywords:** gelatin, Ketoprofen, tannic acid, drug delivery system, wound, wounds healing, anti-inflammatory agent, emulsion

## Abstract

Chronic wounds remain a significant clinical challenge due to persistent inflammation and impaired tissue repair. Anti-inflammatory agents play a pivotal role in wound management by reducing excessive inflammation, preventing further tissue damage, and creating a microenvironment conducive to healing. Among them, Ketoprofen, a non-steroidal anti-inflammatory drug, is effective in modulating inflammation. However, its systemic administration is associated with adverse effects, highlighting the need for localized and controlled delivery systems. Gelatin-based carriers provide important advantages, including biocompatibility, biodegradability, low immunogenicity, cost-effectiveness, and the ease of chemical modification to tailor drug release profiles. In this pioneering study, gelatin-based microspheres crosslinked with tannic acid were developed to achieve sustained topical release of Ketoprofen. The microparticle system was produced through the single water-in-oil emulsification process and optimized by varying homogenization speed, crosslinking time, and molar ratio. Morphological, physicochemical, functional, and biological characterizations were conducted. The optimized formulation yielded spherical microspheres (5–35 µm) with high crosslinking efficiency and a controlled drug release profile over time. COX inhibition assays provided preliminary evidence that released Ketoprofen-retained inhibitory activity under the assay conditions, while cytocompatibility tests supported the short-term compatibility of the system within the tested concentration range. A qualitative wound-model test provided preliminary evidence of powder hydration, film formation, and macroscopic retention. Overall, tannic acid-crosslinked gelatin microspheres represent a biocompatible and promising platform for localized drug delivery of non-steroidal anti-inflammatory in wound management.

## 1. Introduction

Chronic wounds represent a pressing clinical and socioeconomic challenge, characterized by prolonged healing times and a high recurrence rate. Unlike acute wounds, which follow a coordinated timely sequence of physiological events—haemostasis, inflammation, proliferation, and remodeling—chronic wounds exhibit a dysregulated response that often arrests in the inflammatory phase, hampering tissue repair [1,2]. Epidemiological data estimated a global prevalence of 1.67 per 1000 individuals from 2000 to 2022, with venous leg ulcers, diabetic foot ulcers, and pressure ulcers being the most prevalent types [3]. Their high incidence significantly affects the healthcare systems and patients’ quality of life. In the United States alone, the annual cost of treating chronic wounds exceeds USD 10 billion in 2018, while in Europe the economic burden from 2000 to 2008 is estimated at over EUR 12 billion [4,5].

Traditional wound management strategies—including debridement, moist dressings, negative pressure wound therapy, and hyperbaric oxygen therapy—often fail to achieve complete and sustained healing, especially in patients with comorbidities [6,7,8,9,10,11,12,13]. Given the limitations of conventional therapies, scientific research shifted toward the development of advanced wound dressings that do not merely act as passive barriers but actively contribute to the healing process. Advanced dressings are designed to maintain optimal moisture balance, prevent infection, regulate inflammation, and even stimulate tissue regeneration [14]. Central to this paradigm is the incorporation of drug delivery systems (DDSs) that can release therapeutic agents—such as antimicrobials [15,16,17,18,19,20,21], anti-inflammatory compounds [22,23], growth factors [24,25,26,27], and stem cells [28,29,30]—in a sustained and localized manner [31,32,33]. Several carrier platforms have been investigated for localized drug delivery in wound care, including hydrogels, films, electrospun fibrous mats, liposomal and polymeric nanoparticle systems, porous scaffolds, and microspheres [34,35,36,37,38,39]. These systems offer different advantages in terms of moisture retention, wound–bed contact, payload protection or solubilization, structural support, and manufacturing/storage requirements [40]. Hydrogels and films provide intimate contact with the wound surface and support a moist environment, electrospun mats mimic extracellular-matrix structures, nano systems improve drug delivery, and porous scaffolds can combine drug delivery with structural support [41,42,43,44]. In this context, microspheres represent a versatile platform for localized wound drug delivery, combining adaptability to irregular wound geometries with the possibility of modulating drug loading and release through particle properties tuning in terms of composition, size, and crosslinking density [39].

Among the wide spectrum of therapeutic agents, non-steroidal anti-inflammatory drugs (NSAIDs) hold a pivotal role in chronic wound management by downregulating excessive inflammation and creating a microenvironment conducive to repair. Indeed, they have been extensively employed in wound management due to their ability to inhibit the production of prostaglandins via reversible blockade of cyclooxygenase (COX) enzymes [45]. In particular, Ketoprofen (K), a non-selective NSAID of the aryl propionic acid class, showed excellent anti-inflammatory, analgesic, and antipyretic properties [46,47]. Its mechanism of action involves competitive inhibition of both COX-1 and COX-2 isoforms, resulting in reduced prostaglandin synthesis [48]. However, their systemic administration is fraught with side effects, including gastrointestinal ulceration, renal toxicity, and increased cardiovascular risk, especially in vulnerable patients [49,50,51,52,53,54,55]. These issues, combined with the need for frequent dosing due to short plasma half-life, underscored the importance of localized delivery strategies. Topical administration of NSAIDs emerged as a promising alternative, allowing for targeted anti-inflammatory action at the wound site while minimizing systemic exposure. In this context, the design of a safe and effective topical carrier becomes crucial to harness the pharmacological benefits of K while overcoming its clinical limitations.

Gelatin, a natural polymer derived from the partial hydrolysis of type I collagen, emerged as a highly attractive material for the development of DDSs due to its excellent biocompatibility, bioactivity, biodegradability, bioadhesiveness, as well as its ease of handling and manufacturing [56,57,58,59,60,61]. Among gelatin-based DDSs, gelatin microspheres (MSs) have proven to be a versatile carrier for both hydrophilic and hydrophobic drugs [61,62]. Moreover, they enable precise modulation of particle size, surface morphology, and degradation behavior, which are critical parameters for achieving controlled drug release [63,64,65,66,67]. Although MS-based systems are successfully clinically applied for several biomedical applications, such as intra-articular treatments, embolization therapy, and localized chemotherapy [68,69,70,71,72], their use for wound healing remains under-investigated, especially in combination with anti-inflammatory agents. To develop MS and to tune their performances, several types of crosslinking agents have been employed [73]. A combination of chemical (i.e., synthetic: calcium chloride, glutaraldehyde, diisopropylcarbodiimide, dialdehyde carboxymethyl cellulose, formalin, methylenebisacrylamide, formaldehyde; natural: genipin), physical (i.e., heat), and enzymatic (i.e., transglutaminase) crosslinkers were employed [61,74,75,76,77]. Among naturally derived crosslinkers, tannic acid (TA), a plant-derived polyphenol, has attracted growing interest for the development of collagen/gelatin-based substrates [78,79]. TA interacts with gelatin through multiple formulation-dependent mechanisms, including hydrogen bonding between phenolic hydroxyl groups and gelatin polar groups, covalent reactions with nucleophilic amino groups, hydrophobic associations, π–π interactions, and van der Waals forces [78,80]. These interactions may affect the structural integrity and hydration behavior of gelatin-based networks, thereby influencing their degradation and drug-release profiles [78,81,82]. TA has been incorporated into gelatin-based hydrogels, films, and porous matrices because of its polyphenolic structure and ability to stabilize polymer networks [81,82,83]. However, up to date, TA has never been reported as a crosslinking/stabilizing agent for the development of gelatin-based microparticles.

In this context, the aim of the present study is to assess the potential of TA as an innovative crosslinking agent for the production of gelatin-based MS using the single emulsion method. In particular, gelatin-based MSs were conceived to be a dry-sprayable formulation for the localized and controlled K delivery in wound-related settings. The specific contribution of this work lies in the systematic investigation of how homogenization speed, crosslinking time, and TA:PHC molar ratio affect MS morphology, crosslinking degree, swelling, degradation, K encapsulation, release behavior, and preliminary biological performances. MSs were synthesized via a single-step W/O emulsification process. The influence of processing parameters like homogenization speed, crosslinking time, and molar ratio on MS properties was assessed. Several characterization techniques were used to offer an integrated understanding of the physicochemical, functional, and biological features of the system, ensuring its suitability for wound-healing applications. Morphological and size distribution analyses (via optical and electron microscopy) were performed to assess their geometry and uniformity. Chemical and structural properties were investigated through Fourier-transform infrared spectroscopy (FTIR) and crosslinking efficiency assays. Swelling analyses and degradation resistance were performed to evaluate MS behavior in physiological-like conditions and stability, whereas in vitro release studies provided insight into K release kinetics. The preservation of drug activity was confirmed by COX inhibition cell-free assays. Biocompatibility testing was performed to ensure that the developed system did not elicit cytotoxic effects, a prerequisite for their safe application in wound-healing therapies. Lastly, the potential biomedical applicability was assessed through tests on wound models. The system was designed for topical administration in the form of a powder spray, capable of absorbing wound exudate and providing an in situ sustained local drug release in the wound bed. With this approach, the system would enable a prolonged and localized drug release, and offer a biocompatible, non-invasive, and effective strategy for managing inflammation in chronic wounds while reducing the need for systemic NSAID consumption.

## 2. Materials and Methods

### 2.1. Materials

Partially hydrolysed type I collagen (PHC) from equine tendon was provided by Typeone Biomaterials Srl (Calimera, Italy). Distilled water was prepared using a Milli-U10 purification system (Merck Millipore, Darmstadt, Germany). COXs activity inhibition and selectivity were evaluated by the Cayman kit and carried out in accordance with the manufacturer’s instructions. Unless otherwise specified, all chemicals were of analytical grade and purchased from Sigma-Aldrich (Milan, Italy).

### 2.2. Microspheres Synthesis

**Figure 1:**
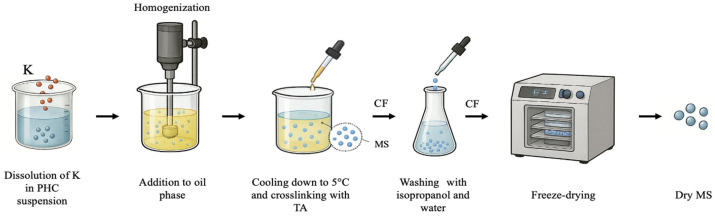
Production process workflow of TA crosslinked MSs loaded with K via W/O single emulsion. CF denotes centrifugation. The schematic illustration was drafted using the AI-assisted design tool FigureLabs (https://figurelabs.ai, accessed on 8 January 2026).

MSs were prepared using a single W/O emulsion method [68,70,84,85,86] (Figure 1). Briefly, 2.5 mL of a 10% (*w*/*v*) PHC solution were preheated to 60 °C to ensure complete dissolution [87]. The PHC solution was added dropwise into preheated sunflower oil (60 °C) under constant stirring at a water/oil ratio of 1:10. The emulsion was obtained by homogenizing the W/O suspension by mean of an IKA T25 Ultra-Turrax homogenizer (IKA-Werke GmbH & Co. KG, Staufen im Breisgau, Germany) at 1000, 3000, and 10,000 rpm for 5–15 min. After the prefixed time, the W/O emulsion was rapidly cooled to 5 °C to induce PHC gelation [88]. Then, crosslinking was done by adding 1 mL of TA solution at different molar ratios to PHC directly into the emulsion for 0.5–4 h. All tested experimental conditions were summarized in Table 1. MSs were recovered by centrifugation (3000 rpm, 5 min, 4 °C), washed with isopropanol (twice), with distilled water (three times), and finally lyophilized for 24 h using a LIO-5P freeze-drier (Cinquepascal s.r.l., Trezzano sul Naviglio, Italy). To obtain drug-loaded microspheres (MSs/K), K powder was incorporated into the PHC solution (K:PHC = 1:7 *w*/*w*) prior to emulsification. Crosslinking time was varied (0.5, 2, and 4 h) to assess its effect on encapsulation efficiency and release [87] (Table 1).

### 2.3. Synthesis Yield

Synthesis yield was calculated as the percentage of dry MS mass over the total mass materials used for their preparation, that were PHC, TA, and, if present, K [89]. The test was repeated three times for each sample type, in triplicate.

### 2.4. Morphological Analysis

The morphology of MSs was observed both in lyophilized and hydrated states. Freeze-dried MS properties were analyzed using an EVO^®^ 40 Scanning Electron Microscope (SEM) (Carl Zeiss Microscopy GmbH, Jena, Germany). About 2–4 mg of freeze-dried samples were mounted on aluminum stubs using carbon tape and observed at an accelerating voltage of 20 kV. MS shape in a hydrated state was observed by means of an Optika B-500 (Optika Srl, Ponteranica, Italy) optical microscope. Approximately 1 mg of dried MS was suspended in 5 mL of distilled water, and a droplet of the suspension was placed on a microscope slide. Images were captured and analyzed using ImageJ software (v 1.54).

### 2.5. Diameters Distribution

The dimensional analysis of MSs was performed in both dry and hydrated MS states. The size distribution of dry MSs was evaluated from SEM images using ImageJ software (National Institutes of Health, Bethesda, MD, USA). Before analysis, each SEM image was calibrated using the corresponding scale bar. For each formulation, at least three representative micrographs acquired at the same magnification were analyzed. Individual, well-defined, and isolated microspheres were manually selected, and their diameters were measured using the Measure tool in ImageJ. At least 200 microspheres were measured for each formulation to ensure a statistically representative particle population. The measured diameters were subsequently used to construct particle size distribution histograms, and the characteristic diameters D10, D50, and D90 were calculated using Microsoft Excel, version 16.97 (Microsoft Corporation, Redmond, WA, USA). These values corresponded to the particle diameters below which 10%, 50%, and 90% of the cumulative sample volume were detected, respectively.

Thereinafter, the dimensional analysis of wet MSs was assessed using a CILAS 1190L particle size analyzer (CILAS, Orléans, France). Approximately 50 mg of MSs were suspended in 5 mL of distilled water and stirred for 6 h at room temperature to ensure full hydration and maximal swelling prior to measurement. During the test, the obscuration level was maintained between 2 and 5%, and the refractive index was set to 1.5, corresponding to the optical properties of gelatin [90]. Each sample was tested in triplicate. The particle size distribution curves were automatically generated by the instrument software based on laser diffraction measurements, which also directly calculated the characteristic particle diameters D10, D50, and D90.

### 2.6. Crosslinking Degree

The degree of crosslinking (CD%) was assessed by quantifying the amount of unreacted TA in the aqueous phase harvested after the first centrifugation step. In particular, after the separation of the supernatant from the MS pellet, 10 mL of distilled water was added to promote stratification and facilitate removal of the residual oil phase. The upper oil layer was carefully discarded, and the aqueous phase—containing unreacted TA—was recovered for analysis. The volume of the aqueous solution was recorded, and the pH was adjusted to 9.0 using 1 M NaOH. Under alkaline conditions, the phenolic groups of TA undergo deprotonation to form phenolate species, resulting in a change in the UV–Vis absorption spectrum in the 300–350 nm region [91]. The absorbance of each sample was measured at λ = 320 nm using a CLARIOstar Plus microplate reader (BMG LABTECH GmbH, Ortenberg, Germany). Quantification of unreacted TA was performed by interpolating the absorbance values on a calibration curve constructed using a standard solution of 1 mg/mL TA at pH 9. The crosslinking degree (CD%) was calculated using the following equation:

(1) $$CD\%=\frac{{TA}_{i}-{TA}_{u}}{{TA}_{i}}\times 100$$

where TA_i_ is the initial amount of TA used for the crosslinking reaction, and TA_u_ is the amount of unreacted TA recovered in the aqueous phase.

### 2.7. Chemical Groups Investigation

Chemical composition was assessed by FT-IR by means of an FT-IR-6300 spectrometer equipped with an ATR module (Jasco GmbH, Pfungstadt, Germany). Few mgs of dry samples of MSs were deposited on the sample holder of the ATR module. Spectra were collected from 4000 to 400 cm^−1^, with a resolution of 4.0 cm^−1^ and 64 scans. Spectra of PHC, TA, K, and sunflower oil were also acquired for comparison. Data were analyzed using OriginPro, version 8.5.0 (OriginLab Corporation, Northampton, MA, USA).

### 2.8. Swelling Degree

The behavior in the aqueous environment of MSs was investigated by calculating the swelling degree under simulated physiological conditions. Approximately 20 mg of lyophilized MSs were dispersed in 1 mL of phosphate-buffered saline (PBS, 0.01 M, pH 7.4) and incubated at 37 °C. At prefixed time points (1, 3, 6, and 24 h), the swollen MSs were separated from the medium by centrifugation at 3000 rpm for 5 min [92,93]. The supernatant was removed, and the MS weight was recorded using an analytical balance. The degree of swelling (SD%) was calculated as:

(2) $$SD\%=\frac{W_{t}-W_{i}\;}{W_{i}}\times 100$$

where W_i_ represents the initial dry weight of the MS and W_t_ the weight of the swollen MS at the respective time point.

### 2.9. Degradation Resistance

The aqueous stability and degradation kinetics of MSs were investigated under physiologically relevant conditions. Lyophilized MSs (10 mg) were resuspended in 1.5 mL of 0.01 M PBS, 0.01% (*w*/*v*) sodium azide and 0.005% (*w*/*v*) polyvinyl alcohol. Samples were incubated at 37 °C under continuous agitation. At predetermined time points, MS degradation resistance was investigated both qualitatively and quantitatively. Morphological changes were assessed by optical microscopy to monitor MS integrity during degradation. Degradation resistance was instead quantitatively estimated by mean of a colorimetric assay. Briefly, samples were centrifuged at 3000 rpm for 5 min, and 50 µL of the supernatant were collected to determine the concentration of solubilized protein using the bicinchoninic acid (BCA) assay (QuantiPro™ BCA Assay Kit, Sigma-Aldrich), strictly following the manufacturer’s instructions. A standard calibration curve was prepared using defined concentrations of PHC (50 µg/mL) in 0.01 M PBS at pH 7.4. Absorbance was measured at λ = 562 nm using an Envision^®^ Multimode Plate Reader (PerkinElmer Inc., Waltham, MA, USA). According to the Lambert–Beer law, the PHC concentration in the incubation medium was esteemed. Then, MS resistance to degradation (DR%) was calculated as:

(3) $$DR\%=\;\left(\frac{M_{0}\;-M_{t}}{M_{0}\;}\right)\;\times \;100$$

where M_0_ is the initial amount of PHC in the MS aliquot and M_t_ is the amount of BCA-detectable PHC solubilized in the supernatant at time t. Each sample was tested in triplicate.

### 2.10. Encapsulation Efficiency

The encapsulation efficiency (EE%) of K in MS/K was spectroscopically esteemed. Specifically, MS/K dry samples were dissolved in 1 M NaOH at a final concentration of 3 mg/mL and maintained under continuous agitation for 24 h to ensure complete degradation and release of the encapsulated drug. The complete degradation of MSs was ensured by a rapid optical microscope investigation. Following incubation, samples were filtered through 0.2 µm syringe filters to remove residual polymeric debris. The absorbance of the filtrate was measured at λ = 284 nm [94]. K concentration was quantified by interpolation from a calibration curve prepared with standard solutions of 1 mg/mL K. To account for possible background absorbance, the same procedure was applied to empty MSs, and their absorbance values were subtracted accordingly. EE% was calculated using the following equation:

(4) $$EE\%=\frac{K_{f}\;}{K_{i}}\times 100$$

where K_i_ is the total amount of K used during MS/K synthesis, and K_f_ is the amount of K detected in the filtrate after MS/K dissolution. Each sample was analyzed in triplicate.

### 2.11. In Vitro Drug Release

The in vitro release profile of K from MS/K was evaluated in physiological-like conditions. About 5 mg of MS/K was incubated in 0.01 M PBS (pH 7.4), 0.01% (*w*/*v*) sodium azide, and 0.005% (*w*/*v*) polyvinyl alcohol at 37 °C under continuous agitation. At selected time points (0.5, 1, 3, 5, and 24 h), aliquots were withdrawn and filtered through 0.2 µm syringe filters to remove residual MS. The absorbance of the filtrates was measured at 284 nm [94]. The concentration of K in each sample was determined by interpolation from a calibration curve generated using K standard solutions (1 mg/mL). To correct for potential background absorbance, empty MS were subjected to the same procedure and their absorbance values subtracted from the corresponding readings. The percentage of drug release (R%) at each time point was calculated using the following equation:

(5) $$R\%=\frac{K_{r}}{K_{e}}\times 100$$

where K_e_ is the total amount of K encapsulated in the MS/K and K_r_ is the cumulative amount of drug released at the specified time point. Each sample was tested in triplicate.

To investigate the K-release mechanism, the mean cumulative release profiles of MS/K-1, MS/K-2, and MS/K-3 were fitted to zero-order, first-order, Higuchi, and Korsmeyer–Peppas models. Zero-order, first-order, and Higuchi models were fitted over the complete experimental interval from 0.5 to 24 h. The Korsmeyer–Peppas model was applied only to the initial release region, considering cumulative release values below 60%, according to the following equation:

(6) $$\frac{M_{t}}{M_{\infty }}={kt}^{n}$$

where Mt/M_∞_ is the fraction of K released at time t, k is the kinetic constant, and n is the apparent release exponent. For spherical matrices, n values close to 0.43 are generally associated with Fickian diffusion, whereas values between 0.43 and 0.85 indicate anomalous transport involving both drug diffusion and polymer relaxation or matrix restructuring [95,96]. Goodness of fit was assessed using the coefficient of determination (R^2^). Since only four, three, and two data points below 60% release were available for MS/K-1, MS/K-2, and MS/K-3, respectively, the Korsmeyer–Peppas analysis was considered preliminary and was not used to assign a single exclusive release mechanism.

### 2.12. Cytocompatibility

The 3-(4,5-dimethylthiazol-2-yl)-2,5-diphenyltetrazolium bromide assay, also known as MTT assay, was employed to evaluate the biocompatibility of MS, MS/K, and K samples using two cell lines, human keratinocytes (HaCaT) and embryonic mouse fibroblasts (NIH/3T3). The MTT assay is a colorimetric method used to assess cellular metabolic activity by measuring the activity of mitochondrial oxidoreductase enzymes. These enzymes reduce the water-soluble MTT to insoluble formazan crystals. Following dissolution in dimethyl sulfoxide (DMSO), the resulting purple-colored formazan can be quantified spectrophotometrically. The amount of formazan is directly proportional to the number of living cells, thereby providing an indication of cell viability and biocompatibility.

In detail, in each well of a 96-well plate, HaCaT and NIH/3T3 cells were seeded at a density of 1 × 10^4^ and 5 × 10^3^ per well, respectively. The multi-well plate was maintained at 37 °C in a humified atmosphere. After overnight incubation, cells were treated with MS, MS/K, and K at different concentrations. After 24 h, the medium was removed and replaced with a serum-free medium containing 2 mg/mL MTT and incubated for 2 h at 37 °C. The MTT reagent was then removed, and the formazan crystals were solubilized using DMSO. The absorbance of the solution was measured at 570 nm on a microplate reader (CLARIOstar Plus, BMG Labtech). Cell viability was assessed using the following formula, where control samples referred to untreated cells.

(7) $$Cell\;viability\;\left(\%\right)=\frac{Absorbance\;samples}{Absorbance\;control\;sample}\times 100$$

### 2.13. Cyclooxygenase Activity Inhibition and Selectivity Determination

The cyclooxygenase activity inhibition ability of MS/K was in vitro assessed by evaluating their ability to inhibit COX enzymes, with the aim of indirectly determining the effectiveness of K released over time. MS/K, MS, K, and TA were incubated at 37 °C in DMSO at a final concentration of 1 mg/mL for 1, 5, and 24 h. This time-course approach allowed for comparison of the inhibitory activity of K released from the MS/K with that of the free drug and of the other individual components. Following incubation, samples were analyzed for their inhibitory effect on ovine COX-1 and human recombinant COX-2 using a cell-free colorimetric COX activity inhibition assay (Cayman Chemical, Cat. No. 7601050, Ann Arbor, MI, USA), according to the manufacturer’s instructions [97]. Absorbance measurements were carried out using a Victor^3^ microplate reader (PerkinElmer, Inc., Waltham, MA, USA). Data were analyzed using GraphPad Prism software, version 10 (GraphPad Software Inc., San Diego, CA, USA). Each sample was tested in triplicate.

### 2.14. Evaluation of MS Adhesion to Wound Model by Dry Spray

The MS/K were formulated as a dry sprayable adhesive powder for direct application onto chronic wound surfaces. A wound model was produced according to an in vitro method described for the characterization of topical formulations [98]. Briefly, a 4% *w*/*v* gelatin solution was prepared by dissolving gelatin in distilled water at 50 °C and poured into Petri dishes. Once gelled, a wound area of about 1 cm in diameter was created by scratching the surface of the gelatin to simulate a superficial lesion. Approximately 0.2 mL of Simulated Wound Fluid (SWF) was then casted into the simulated wound area to mimic the moist environment of a chronic wound. The SWF was formulated to resemble wound exudate and was composed of 0.64% sodium chloride, 0.024% calcium chloride, 0.02% potassium chloride, 0.0048% magnesium chloride, 0.018% potassium phosphate, 0.17% sodium bicarbonate, and 3.4% bovine serum albumin [99]. The lyophilized MS/K were loaded into a non-pressurized spray bottle for dry powders, without any added excipients. Then, spraying of about 5 mg of MS/K (equivalent to 10 erogations) was performed from a fixed distance of 2–3 cm onto the wound site. This assay was designed as a preliminary qualitative assessment. Macroscopic retention was evaluated by visual observation after hydration, inversion of the wound model, and scratching of the formed layer; no quantitative adhesion-force measurements were performed.

### 2.15. Statistical Analysis

Data are presented as mean ± standard deviation (SD). Comparisons between two groups were performed using the unpaired Student’s *t*-test. Comparisons among three or more groups were performed using one-way analysis of variance (ANOVA), followed by Tukey’s post-hoc test where appropriate. For multiple planned pairwise comparisons, *p*-values were adjusted using the Holm method. Differences were considered statistically significant at *p* < 0.05.

## 3. Results

### 3.1. Morphological Analysis

**Figure 2:**
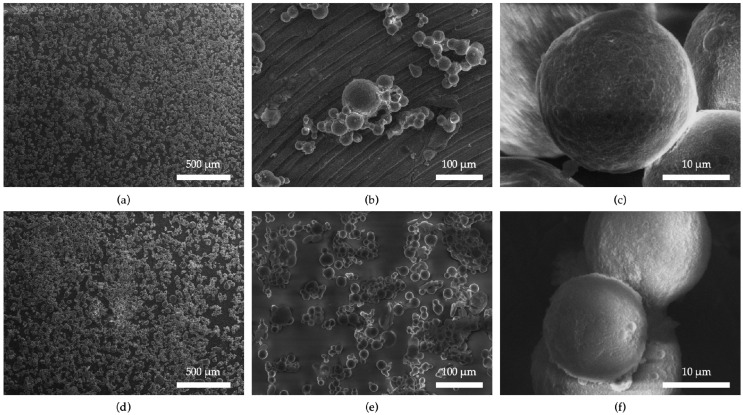
Representative SEM micrographs of MSs at 100× (**a**) and 500× (**b**) magnifications and of MS/K at 100× (**d**) and 500× (**e**) magnifications. Detailed view of MSs (**c**) and MS/K (**f**) surfaces at 7000× magnification.

SEM was performed to investigate the morphology of the synthesized MSs, including particle shape, surface texture, and size distribution. SEM observations confirmed that the pointed-out synthesis protocol successfully allowed to produce gelatin MS with a predominantly spherical morphology (Figure 2a). The single-step W/O emulsification process was found to be effective in generating particles with diameters that fall in the micrometric range (10–40 μm). A low level of aggregation was observed, regardless the adopted protocol (Figure 2b). The surface features appeared to be quite smooth (Figure 2c). The morphology of MSs aligns with previous reports on gelatin-based MS prepared under comparable conditions that were crosslinked with other chemical crosslinker, such as glutaraldehyde or methylenebisacrylamide [100,101].

**Figure 3:**
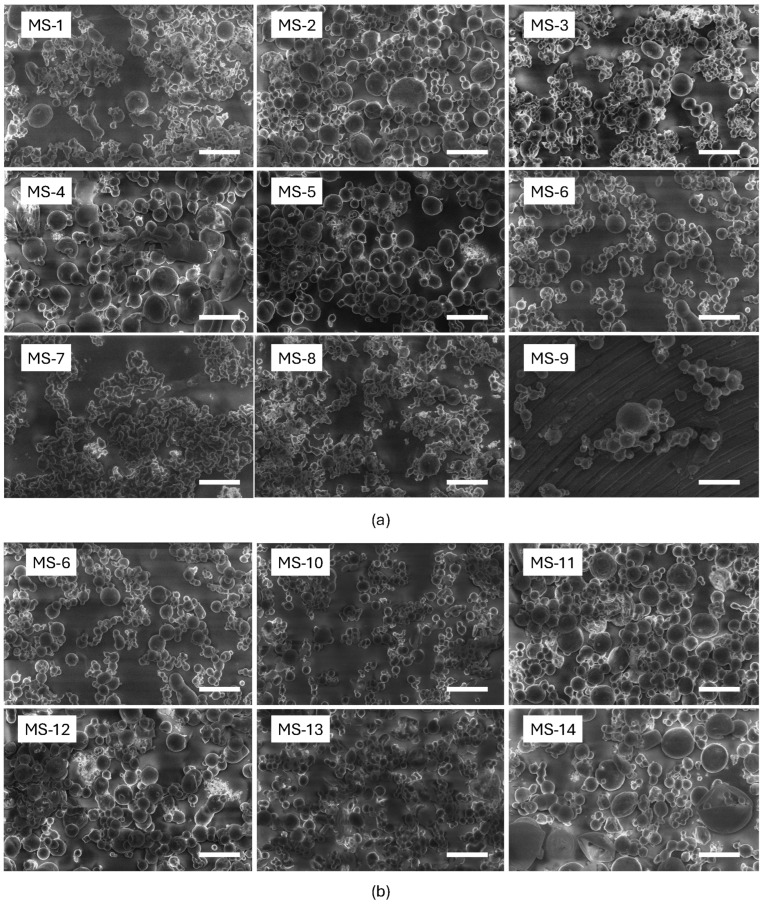
Representative SEM images of empty MSs at varying homogenization (**a**) and crosslinking (**b**) parameters. Scale bar: 100 μm. Magnification: 500×.

The influence of processing parameters on MS morphology was also investigated. SEM micrographs of all conditions were reported in Figure 3. The MS surface was found to be unaffected by processing variables, suggesting that surface topology is primarily governed by intrinsic physicochemical attributes of the gelatin matrix—such as viscosity and chain entanglement degree [102]. Homogenization speed and time were found to strongly influence particle shape. Formulations emulsified at 1000 rpm and 3000 rpm for 10–15 min (MS-2, MS-3, MS-5, MS-6) exhibited a more uniform and well-defined round-like shape, while shorter emulsification times (5 min) led to irregular MSs with a broader size distribution (MS-1, MS-4), likely due to insufficient droplet stabilization during the early emulsification phase. Notably, samples processed at 3000 rpm for 15 min (MS-6) showed the most homogeneous morphology, characterized by consistent particle sizes and minimal deformation.

In contrast, crosslinking parameters significantly modulated particle architecture and organization. Extended crosslinking times (4 h) yielded MSs with a reduced aggregation degree, whereas shorter crosslinking times (0.5–2 h) led to increased MS aggregation and surface bridging. These phenomena may result from incomplete or insufficient network stabilization in the selected reaction time, leading to a partial coalescence of adjacent droplets [103,104,105,106,107]. No effect of the crosslinking concentration on MS morphology was observed.

Drug incorporation had no notable effect on MS surface morphology (Figure 2d–f). Indeed, MS/K surface was found to comparable to that one of unloaded formulations. No effect of the crosslinking time on MS/K morphology was observed.

### 3.2. Synthesis Yield

The synthesis protocol of MSs was found to yield about 20% to 45% of dry MSs, with notable variability linked to tested processing parameters (Table 2). The nine formulations prepared under different homogenization speeds and times were compared as independent processing conditions by one-way ANOVA. A statistically significant difference in synthesis yield was observed among MS-1 to MS-9 [F(8,18) = 6.510, *p* < 0.001]. Tukey’s multiple-comparison test showed that MS-9, prepared at 10,000 rpm for 15 min had a significantly higher yield than MS-1, MS-4, and MS-7 (p_adj_ = 0.006, p_adj_ < 0.001, and p_adj_ = 0.006, respectively). MS-8, prepared at 10,000 rpm for 10 min, also showed significantly higher yields than MS-1, MS-4, and MS-7 (p_adj_ = 0.039, p_adj_ = 0.003, and p_adj_ = 0.039, respectively). Notably, the highest yield was reached by MS-9 (45 ± 4%), where homogenization speed and time were both maximized. However, the mechanical advantage of high shear may be partially offset by droplet fragmentation or emulsion destabilization [108]. Therefore, although the maximum yield was achieved at 10,000 rpm, the best MS morphology was obtained at a homogenization speed of 3000 rpm and a homogenization time of 15 min, corresponding to MS-6.

Thus, the effect of crosslinking conditions was investigated in terms of TA:PHC ratio and reaction time. The TA:PHC ratio had a comparatively modest effect on yield. At matched crosslinking times, formulations with a 1:4 ratio (MS-12, MS-13, MS-14) showed no statistically significant differences in yield compared to their 1:2 counterparts after Holm correction (p_adj_ > 0.05), suggesting that the investigated variation in TA:PHC ratio did not consistently enhance particle formation and recovery. On the contrary, crosslinking time exerted a more pronounced influence. At a TA:PHC ratio of 1:2, one-way ANOVA showed a significant effect of crosslinking time on yield [F(2,6) = 13.345, *p* = 0.006]. Tukey’s multiple-comparison test showed that the formulation crosslinked for 4 h had a significantly higher yield than those crosslinked for 2 h and 0.5 h (p_adj_ = 0.005 p_adj_ = 0.045, respectively), whereas no statistically significant difference was observed between 0.5 and 2 h (p_adj_ = 0.201). Similarly, at a TA:PHC ratio of 1:4, one-way ANOVA showed a significant effect of crosslinking time [F(2,6) = 20.793, *p* = 0.002]. The formulation crosslinked for 4 h showed a significantly higher yield than those crosslinked for 0.5 and 2 h (p_adj_ = 0.002 and p_adj_ = 0.012, respectively), whereas no statistically significant difference was observed between 0.5 and 2 h (p_adj_ = 0.201). These results suggest the need for longer reaction times to better stabilize the system and improve particle recovery. A similar descriptive trend was observed in drug-loaded samples, with MS/K-2 and MS/K-3 yielding less than MS/K-1 (31 ± 2%), which was exposed to the crosslinker for a longer time. However, this difference was not statistically significant by one-way ANOVA [F(2,6) h = 3.828, *p* = 0.085].

### 3.3. Diameters Distribution

**Figure 4:**
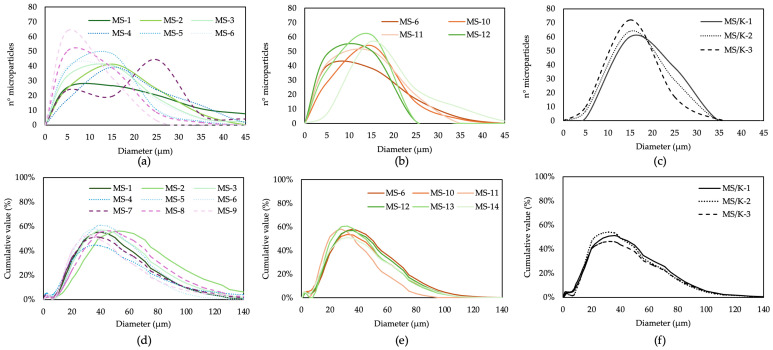
Size distribution of dry MS esteemed from SEM images in relation to homogenization parameters (**a**), the amount of crosslinking agent and crosslinking time (**b**), the presence of incapsulating agent (**c**). Size distribution of wet MSs calculated by laser diffraction in relation to homogenization parameters (**d**), the amount of crosslinking agent and crosslinking time (**e**), the presence of incapsulating agent (**f**).

Diameters distribution of MSs was investigated on MSs in both dry and hydrated states (Figure 4). Diameters distribution in the dry state was estimated by SEM analysis, while that in hydrated state by laser diffraction analysis.

Diameters distribution in the dry state is reported in Figure 4a–c. Generally, as calculated from SEM micrographs, dry MS size was found to be in the micron range, with D10, D50, and D90 values of about 1–3 μm, 5–15 μm, and 15–40 μm respectively (Table S1). Marked was the influence of processing conditions. Under a constant TA:PHC ratio of 1:2 (Figure 4a), the increment of the homogenization speed from 1000 rpm (MS-1, MS-2, MS-3) to 3000 rpm (MS-4, MS-5, MS-6) resulted in a slight reduction of the particle size. The further increase of homogenization speed to 10,000 rpm allowed for a marked reduction in particle size, suggesting how homogenization speed is inversely proportional to the MS size. Under constant homogenization speed, homogenization time promoted narrower size distributions in all cases [109,110].

All these considerations coupled with results of other analyses allowed to identify MS-6 as the best experimental condition. Thus, the effects of crosslinking time and TA:PHC ratio were further assessed in MS-10 to MS-14 (Figure 4b). Keeping emulsification constant (3000 rpm, 15 min), a decrease in crosslinking time led to broader distributions and increased particle fragmentation. Decreasing the TA:PHC ratio from 1:2 to 1:4 (MS-12 to MS-14) further amplified this effect, confirming that reduced crosslinker density facilitates network fragmentation during emulsification [111,112].

Lastly, MS/K exhibited a distinct and narrower distribution (Figure 4c), characterized by a sharp peak at 15 µm. Contrarily of what was observed for empty MSs, crosslinking time did not influence MS size distribution. These results suggested that K presence could lead to earlier MS stabilization and reduces their size heterogeneity [113].

The evaluation of MS behavior in physiological-like conditions (hydrated state) rather than in dry conditions (freeze-dried MSs) is of fundamental importance for the understanding of their performance in vivo. Indeed, the hydrated MS size distribution directly correlates with swelling dynamics, drug release kinetics, and tissue interactions, providing more relevant insights compared to measurements in the dry state. Diameters distribution in physiological-like conditions is reported in Figure 4d–f and Table S2. As expected, hydrated MSs exhibited larger mean diameters, reflecting the intrinsic swelling behavior of the gelatin-based system when exposed to aqueous media. In particular, MS displayed diameters between 20 and 80 μm. Across all samples, the dominant particle range remained between 30 and 40 μm (about 50%), confirming morphological consistency despite variations in swelling profiles.

### 3.4. Crosslinking Degree

The CD% was determined via colorimetric assay for both MSs and MS/K and is reported in Table 2. Under fixed chemical conditions (TA:PHC = 1:2; crosslinking time = 4 h), variations in homogenization speed, and emulsification time (MS-1 to MS-9) resulted in only modest fluctuations in CD%, with values ranging from 94.8% to 97.1%. One-way ANOVA showed no significant differences among these formulations [F(8,18) = 0.485, *p* = 0.851], indicating that processing conditions did not substantially affect crosslinking extent when chemical parameters were kept constant. In contrast, crosslinking time emerged as a critical determinant. At constant emulsification conditions (3000 rpm, 15 min), CD% increased significantly with longer reaction durations at both the 1:2 [F(2,6) = 15.530, *p* = 0.004] and 1:4 [F(2,6) = 22.718, *p* = 0.002] ratios. Tukey’s multiple-comparison test showed that, at the 1:2 ratio, the formulation crosslinked for 4 h had a significantly higher CD% than that crosslinked for 0.5 h (*p_adj_* = 0.003), whereas the other comparisons were not statistically significant. At the 1:4 ratio, formulations crosslinked for 2 h and 4 h showed significantly higher CD% values than that crosslinked for 0.5 h (both *p_adj_* = 0.003), whereas no significant difference was observed between 2 h and 4 h (*p_adj_* = 0.998).

The effect of TA:PHC ratio was less pronounced than that of crosslinking time. No statistically significant differences were observed between the two ratios at 0.5 h or 4 h after Holm correction (*p_adj_* > 0.05). However, at 2 h, the 1:4 formulation showed a significantly higher CD% than the corresponding 1:2 formulation (*p_adj_* = 0.021). These results support the conclusion that longer crosslinking times promote denser networks, enhancing matrix stability and polymer consolidation [114].

The crosslinking density was also investigated in MS/K to assess whether drug incorporation affected the crosslinking process. MS/K exhibited CD% values ranging from 90.9% to 95.8%, consistent with those of their unloaded counterparts (MS-6, MS-10, MS-14). No statistically significant differences were observed after Holm correction (MS-6 vs. MS/K-1, *p_adj_* = 0.900; MS-10 vs. MS/K-2, *p_adj_* = 0.287; MS-14 vs. MS/K-3, *p_adj_* = 0.277), confirming that K incorporation did not adversely affect crosslinking efficiency.

### 3.5. Chemical Groups Investigation

**Figure 5:**
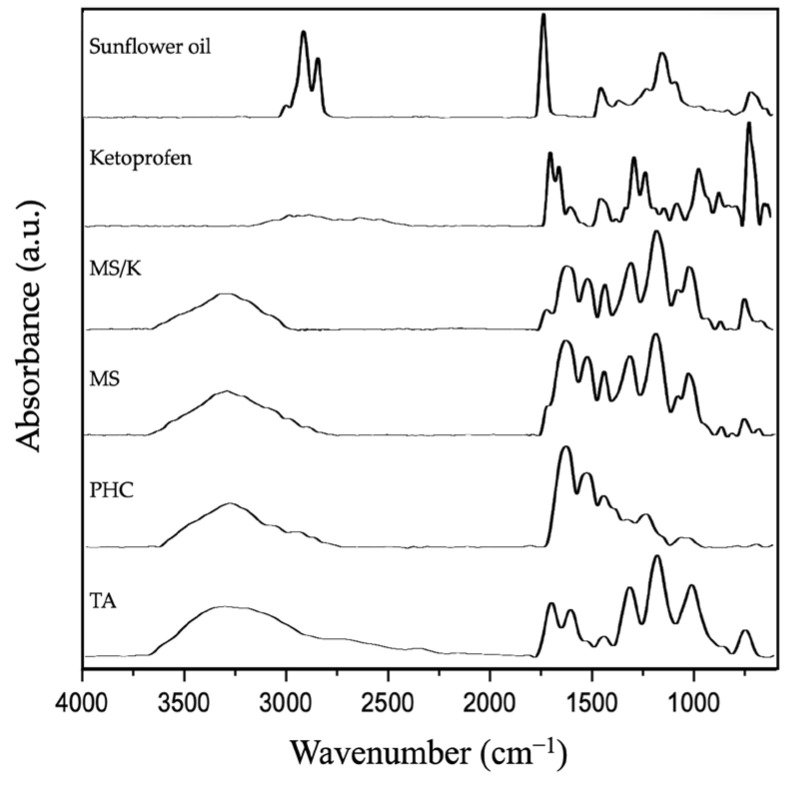
Representative FT-IR spectra of MSs, MS/K, PHC, TA, sunflower oil, and K.

FT-IR spectroscopy was used to investigate the chemical composition and intermolecular interactions of MS components. Raw material spectra (PHC, TA, and K) were acquired and compared with those of MSs and MS/K to more easily attribute peaks and investigate the influence of processing parameters and the effectiveness of drug incorporation. All spectra are reported in Figure 5. The spectrum of PHC exhibited characteristic protein absorption bands. Amide I (C=O stretching) at 1629 cm^−1^, amide II (N–H bending) at 1518 cm^−1^, and amide III (C–N stretching) at 1232 cm^−1^, Amide A (N–H stretching) at 3280 cm^−1^, and Amide B (N–H stretching) at 3058 cm^−1^ were present, in full agreement with the literature [115]. The spectrum of TA was found to be characterized by a broad O–H stretching band centered at 3327 cm^−1^, a C=O band at 1696 cm^−1^, and intense aromatic C=C absorptions between 1600 and 1400 cm^−1^. A prominent C–O stretching band was also observed near 1250 cm^−1^, consistent with its polyphenolic structure [116]. The distortion vibration of C=C in benzene rings was found at 754 cm^−1^. The spectrum of sunflower oil showed prominent aliphatic C–H stretching vibrations near 2920 and 2850 cm^−1^, and ester-related C=O and C–O stretching bands around 1740 cm^−1^ and 1160–1100 cm^−1^, respectively [117]. The FT-IR spectrum of K revealed strong carbonyl stretching bands at 1690 cm^−1^ (carboxyl C=O) and 1646 cm^−1^ (ketone C=O), as well as multiple sharp bands between 1300 and 1000 cm^−1^ associated with C–O stretching of carboxylic-acid and aromatic vibration, consistent with previous studies on aryl-propionic acid NSAIDs [118].

Spectra of all MS types were acquired. However, no differences in peak number or position were observed among the formulations. Therefore, Figure 5 reports only one representative condition (MS-6). The MS spectrum was characterized by contributions from both TA and PHC. Some TA bands overlapped with those of PHC, and small shifts were observed. Specifically, the amide I band shifted from 1629 cm^−1^ (PHC) to 1631 cm^−1^, while the amide II band shifted from 1518 cm^−1^ to 1513 cm^−1^. These shifts indicate changes in the local molecular environment of PHC following TA incorporation and are consistent with the establishment of PHC–TA interactions. Hydrogen bonding between the phenolic hydroxyl groups of TA and polar groups of PHC is likely to contribute to network stabilization. Hydrophobic association, possible π–π interactions involving aromatic moieties, and van der Waals forces may also contribute to molecular assembly. Depending on formulation conditions, covalent reactions between phenolic groups and nucleophilic amino groups cannot be excluded. However, ATR-FTIR does not allow the individual contributions of these interactions to be directly identified or quantified. The observed spectral changes should therefore be interpreted as evidence of PHC–TA interactions rather than proof of a single crosslinking mechanism [78,80,81,82,119,120]. However, it could be clearly assessed that shifts observed in the amide I and amide II bands of the MS relative to uncrosslinked PHC provide clear evidence of changes in the molecular environment of the gelatin chains following treatment with TA. Such spectral changes are consistent with the formation of intermolecular interactions between PHC and TA during the crosslinking process. The TA peaks’ presence in MSs was not due to a physical entrapment of TA in the PHC matrix because, without crosslinkers, PHC is not able to self-organize in MSs. Indeed, in absence of a crosslinking agent, PHC-based MSs could not be successfully formed or recovered following the emulsion process (see Figure S1, reporting our preliminary attempts to prepare MS without any crosslinker). The absence of peaks attributable to sunflower oil confirmed the effective removal of oil residues during the purification step.

In MS/K, the FT-IR spectrum retained the main bands of the MS polymeric network, and additional signals corresponding to K loading were observed. A new peak at 1734 cm^−1^, attributable to the C=O stretch of the ketonic group of K, was clearly detected. Furthermore, the region between 1250 and 1050 cm^−1^ showed intensified C–O stretching bands typical of ester and ether groups. These findings suggested that the drug could be incorporated inside the MS structure since K peaks visualization was hindered by the strong MS components signal. The absence of major new bands or marked spectral shifts is compatible with the physical incorporation of K within the MS matrix.

### 3.6. Swelling Degree

**Figure 6:**
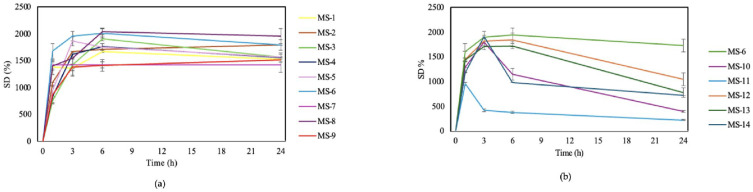
Swelling degree of MSs in relation to homogenization parameters (**a**) and crosslinker concentration (**b**) during time. Reported values represent the mean ± SD (*n* = 3).

The swelling behaviour of MSs was evaluated in relation to key synthesis parameters and is reported in Figure 6. Under constant crosslinking conditions (TA:PHC = 1:2; 4 h), formulations MS-1 to MS-9 achieved high hydration levels, with maximum swelling degrees (SD%) ranging from approximately 1500% to 2100% within 3–6 h (Figure 6a) [121]. One-way ANOVA performed on the final SD% values showed statistically significant differences among the nine formulations [F(8,18) = 10.719, *p* < 0.001]. Tukey’s multiple-comparison test showed that MS-8 exhibited significantly higher final SD% than MS-1, MS-3, MS-4, MS-5, MS-7, and MS-9 (all p_adj_ ≤ 0.003). Samples prepared under higher shear and extended emulsification time, such as MS-6 and MS-9, exhibited delayed swelling kinetics, likely due to their reduced particle size and increased surface area, which may hinder rapid water absorption [122,123]. By contrast, crosslinking time exerted a pronounced effect on the swelling behavior (Figure 6b). With emulsification conditions fixed at 3000 rpm for 15 min, SD% at 24 h increased significantly with crosslinking time. At a TA:PHC ratio of 1:2, one-way ANOVA showed a significant effect of crosslinking time on final SD% [F(2,6) = 373.891, *p* < 0.001]. MS-6, crosslinked for 4 h, showed the highest apparent swelling (1731 ± 127%), significantly exceeding MS-10, crosslinked for 2 h (397 ± 14%; p_adj_ < 0.001), and MS-11, crosslinked for 0.5 h (223 ± 10%; p_adj_ < 0.001). The difference between MS-10 and MS-11 was not statistically significant after correction (p_adj_ = 0.063). However, these data should be interpreted as apparent swelling values, because hydration and partial matrix degradation may occur simultaneously. Poorly crosslinked MS partially degraded during 24-h incubation (see Section 3.7), reducing pellet recovery after centrifugation and lowering the measured Wt. Thus, the higher apparent SD% observed after 4 h of crosslinking likely reflects greater matrix integrity and retention of swollen material, rather than a simple increase in intrinsic mesh expansion.

As it regards the effect of crosslinker content, the TA:PHC ratio influenced the apparent swelling behaviour, although the direction of this effect depended on crosslinking time. At 0.5 h and 2 h, formulations prepared at a TA:PHC ratio of 1:4 showed significantly higher final SD% than the corresponding 1:2 formulations [t(4) = −7.735, p_adj_ = 0.005; and t(4) = −6.917, p_adj_ = 0.005, respectively]. Conversely, under extended reaction time, MS-6 showed significantly higher final SD% than MS-12 (1731 ± 127% vs. 1045 ± 97%; t(4) = 7.451, p_adj_ = 0.005). Therefore, the TA:PHC ratio affected the swelling behaviour, with a reduction of hydration degree with increasing crosslinker content and time.

**Figure 7:**
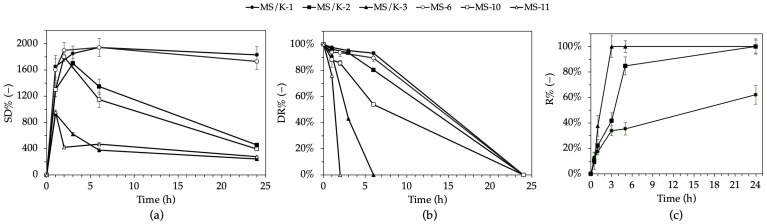
MS/K properties in comparison with their respective empty MSs in terms of SD% (**a**), D% (**b**), and R% (**c**), evaluated during time in physiological-like conditions. Reported values represent the mean ± SD (*n* = 3).

K-loaded formulations (MS/K-1 to MS/K-3) followed similar trends (Figure 7a). MS/K-1, crosslinked for 4 h, reached an SD% of 1942 ± 140% and was statistically comparable to MS-6 after Holm correction (p_adj_ = 1.000), indicating that drug loading did not impair network expansion under prolonged crosslinking conditions. MS/K-2, crosslinked for 2 h, showed a higher mean SD% than MS-10 (1345 ± 116% vs. 1145 ± 116%), but this difference was not statistically significant after correction (p_adj_ = 0.205). Conversely, MS/K-3, crosslinked for 0.5 h, exhibited significantly lower SD% than MS-14 (379 ± 25% vs. 982 ± 109%; p_adj_ = 0.002), suggesting that drug–polymer interactions may influence apparent swelling behavior when the crosslinking time is limited.

### 3.7. Degradation Resistance

**Figure 8:**
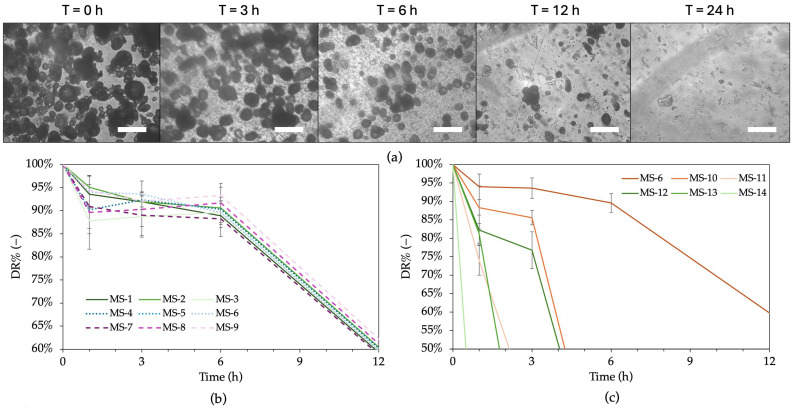
Representative optical microscopy images of MSs during in vitro degradation over time (scale bar: 50 μm; magnification: 20×) (**a**), and DR% profile of MS in relation to homogenization (**b**) and crosslinking parameters (**c**). Reported values represent the mean ± SD (*n* = 3).

The degradation behavior of MSs was evaluated under physiological-like conditions over 24 h both qualitatively and quantitatively (Figure 8). MSs were firstly observed by optical microscopy at predefined time points to assess the presence of structurally intact particles. Then, the degradation resistance was quantified through a colorimetric assay by measuring BCA-detectable PHC solubilized in the supernatant. Figure 8a shows a representative degradation time course together with quantitative data. All samples initially exhibited high degradation resistance, followed by a progressive decrease over time. A complete structural integrity (DR% = 0% after 15 min), followed by a slight degradation after 1 h was detected. At 24 h, all MS types reached DR % values close to 0%, indicating extensive PHC solubilization under the tested conditions, regardless of processing conditions.

Under fixed chemical conditions (TA:PHC = 1:2; 4 h crosslinking), formulations MS-1 through MS-9 maintained high degradation resistance up to 6 h, with D% values ranging from approximately 88% to 93% (Figure 8b). One-way ANOVA performed on DR% values measured at 6 h showed no statistically significant differences among these formulations [F(8,18) = 0.662, *p* = 0.718], confirming that variations in homogenization speed and emulsification time did not significantly influence short-term degradation resistance.

In contrast, crosslinking time emerged as a critical determinant of degradation resistance (Figure 8c). When emulsification parameters were held constant at 3000 rpm for 15 min, shorter crosslinking times significantly accelerated PHC solubilization. At a TA:PHC ratio of 1:2, one-way ANOVA showed significant differences in DR% at 3 h among formulations crosslinked for different durations [F(2,6) = 2046.790, *p* < 0.001]. MS-6, crosslinked for 4 h, retained high degradation resistance (93.6 ± 2.8%), whereas MS-10, crosslinked for 2 h, and MS-11, crosslinked for 0.5 h, showed DR% values of 85.6 ± 2.0% and 0.0%, respectively. Tukey’s multiple-comparison test showed that MS-6 had significantly higher DR% values than both MS-10 and MS-11 (p_adj_ = 0.006 and p_adj_ < 0.001, respectively). MS-10 also showed significantly higher degradation resistance than MS-11 (p_adj_ < 0.001).

A similar trend was observed at a TA:PHC ratio of 1:4. MS-14, crosslinked for 0.5 h, showed complete loss of degradation resistance after 1 h, confirming the pronounced influence of insufficient crosslinking time. At 1 h, one-way ANOVA showed significant differences among MS-14, MS-13, and MS-12 [F(2,6) = 869.598, *p* < 0.001]. MS-13 and MS-12, crosslinked for 2 h and 4 h, respectively, showed significantly higher DR% values than MS-14 (both p_adj_ < 0.001), whereas no significant difference was observed between MS-13 and MS-12 (p_adj_ = 0.918). These findings support that extended reaction times enhance network density, thereby improving resistance to PHC solubilization [122,123,124].

The role of crosslinker concentration was further evaluated (Figure 8c). MS crosslinked for 4 h (MS-6 and MS-12) showed DR% values of 94.0 ± 3.4% and 82.2 ± 4.0% after 1 h of incubation, respectively. This difference remained statistically significant after Holm correction [t(4) = 3.850, p_adj_ = 0.037]. By comparing MS-10 and MS-13, both crosslinked for 2 h, no statistically significant difference was observed after Holm correction [t(4) = 1.905, p_adj_ = 0.129], although MS-13 showed a lower mean D% value than MS-10. Instead, MS crosslinked for 0.5 h showed rapid loss of degradation resistance, with MS-11 and MS-14 reaching DR% values close to 0% at early time points.

Drug-loaded MS (MS/K-1 to MS/K-3) showed degradation-resistance profiles that depended on crosslinking time (Figure 7b). MS/K-1 retained 93.2 ± 1.3% degradation resistance at 6 h, with no statistically significant difference compared with MS-6 after Holm correction (p_adj_ = 0.094). MS/K-2 maintained a DR% value of 89.9 ± 1.3% at 6 h, whereas the corresponding unloaded formulation showed complete loss of degradation resistance at the same time point (MS-10). Similarly, MS/K-3 maintained a DR% value of 92.0 ± 1.1% at 1 h, whereas the corresponding unloaded formulation had already reached a DR% value close to 0% (MS-11). Thus, K incorporation did not impair degradation resistance under prolonged crosslinking conditions and appeared to improve resistance to PHC solubilization under shorter crosslinking times, possibly through hydrophobic or hydrogen-bonding interactions [124].

### 3.8. Encapsulation Efficiency and Release Kinetics of K from MS/K

The EE% of K and its release kinetics from MS/K were spectroscopically evaluated. All three formulations successfully entrapped K with an EE% of 38.9 ± 1.7% for MS/K-1, 22.5 ± 4.4% for MS/K-2, and 15.2 ± 1.4% for MS/K-3. One-way ANOVA showed that crosslinking time significantly affected EE% [F(2,6) = 54.767, *p* < 0.001], with significant differences among all formulations according to Tukey’s post-hoc test. Longer crosslinking times promoted the formation of a denser and more cohesive polymer matrix, enhancing drug retention by minimizing leakage through the polymeric matrix during washing steps, whereas shorter reaction times led to less structured networks that were more susceptible to drug loss.

The release kinetics of K from MS/K were reported in Figure 7c, with a crosslinking time dependent behavior significant from 1 h onward [1 h: F(2,6) = 10.383, *p* = 0.011; 3 h: F(2,6) = 92.589, *p* < 0.001; 5 h: F(2,6) = 106.459, *p* < 0.001; 24 h: F(2,6) = 37.375, *p* < 0.001]. Poorly crosslinked MS/K-3 showed a burst release, with 37.7 ± 3.5% of K released after 1 h and almost complete release within 3 h. In contrast, longer crosslinking times allowed better release control, with approximately +15% drug retention in MS/K-2 and +25% drug retention in MS/K-1 after 1 h. Thus, MS/K-1 exhibited the most sustained profile, reaching 62.1 ± 1.4% cumulative release after 24 h, whereas MS/K-2 released 84.7 ± 2.0% of the drug within 5 h and approached complete release after 24 h. To further characterize the release behavior, the experimental release profiles of MS/K were fitted to zero-order, first-order, Higuchi, and Korsmeyer–Peppas models (Table 3). For MS/K-1, the Higuchi model provided the best whole-profile fit (R^2^ = 0.884), and the Korsmeyer–Peppas exponent *n* = 0.440 was close to the theoretical Fickian limit for spherical systems, supporting a mainly diffusion-driven release through the hydrated PHC matrix [125,126]. For MS/K-2, the first-order model showed the best whole-profile fit (R^2^ = 0.950), while *n* = 0.675 suggested a possible contribution of anomalous transport involving both diffusion and matrix relaxation. However, this interpretation remains preliminary because only three points were available below 60% release [125]. MS/K-3 was also best described by the first-order model (R^2^ = 0.916), but its burst-release profile and limited number of early-release points prevented reliable Korsmeyer–Peppas classification. Overall, increasing crosslinking time shifted K release from a burst profile toward a more sustained, mainly diffusion-controlled behavior, without supporting the attribution of a single exclusive release mechanism to all formulations.

### 3.9. Cytocompatibility

**Figure 9:**
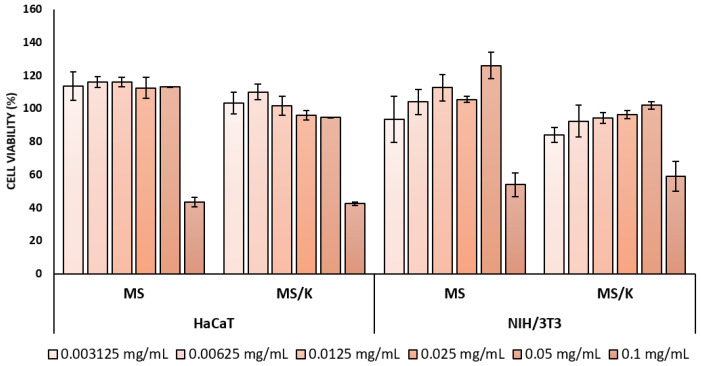
Percentage of cell viability of HaCaT and NIH/3T3 cell lines after 24 h incubation with MS and MS/K at different concentrations, estimated through MTT assay. Reported values represent the mean ± SD (*n* = 3).

The colorimetric MTT assay was used to evaluate and compare the cytocompatibility of MS, MS/K, and K after 24 h incubation with HaCaT and NIH/3T3 cell lines. The effects of MS and MS/K were assessed at concentrations ranging from 3 to 0.100 µg/mL. The results showed the optimal biocompatibility of both MS and MS/K in both cell lines up to 0.05 mg/mL. The cellular viability dropped below 60% at the highest concentration (0.1 mg/mL), likely because of the massive presence of particles that tend to settle down over time, thus covering the seeded cells. (Figure 9).

The free drug was assayed as well. The tested concentrations of K were selected according to the estimated EE%. The results reported in the Supplementary Information (Figure S2), evidence the optimal biocompatibility (higher than 80%) for all the tested concentrations.

### 3.10. Cyclooxygenase Activity Inhibition and Selectivity Determination

**Figure 10:**
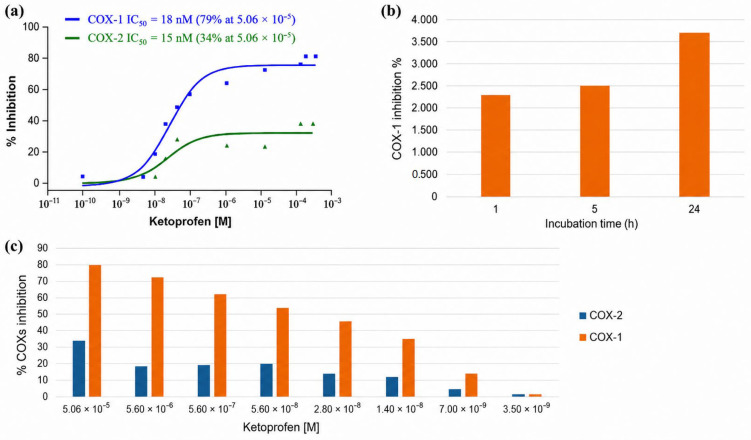
Inhibitory activity values of K against COXs (**a**); percentage of COX-1 inhibition for MS/K-1 over time (**b**); percentage inhibition of COXs by K relative to concentration (**c**). Reported values represent the mean ± SD (*n* = 3).

The inhibitory activity of K toward cyclooxygenase isoforms was preliminarily evaluated in order to properly interpret the biological contribution of the encapsulated drug. The maximum K concentration encapsulated within the formulations, as determined from encapsulation efficiency studies, was 5.06 × 10^−5^ M. At this concentration, K produced a maximal inhibition of 79% against COX-1 and 34% against COX-2 (Figure 10a). These data further confirm the non-selective profile of K, characterized by a markedly higher functional inhibition of COX-1 compared to COX-2 at pharmacologically relevant concentrations. Although comparable IC_50_ values were determined for the two isoforms, the substantially lower maximal inhibition observed for COX-2 indicates reduced efficacy toward this isoenzyme under the experimental conditions employed. Importantly, higher concentrations used during assay optimization (up to 100-fold the encapsulated concentration) were selected exclusively for IC_50_ determination and do not reflect the effective Ketoprofen levels present in the formulations. Therefore, the inhibition percentages observed at 5.06 × 10^−5^ M represent the most biologically relevant values for evaluating the contribution of K to the overall COX inhibitory activity of the formulations [127]. Regarding TA’s COX inhibitory capacity at the maximum concentration used in the formulations, it was found to be completely ineffective in inhibiting COX activity (Table S3).

To verify the percentage of inhibition of MSs and MS/K, each sample was resuspended in DMSO at a concentration of 1 mg/mL for different times (1, 5, and 24 h) at 37 °C, to simulate the release of K over time. After incubation, samples were subjected to the COX inhibition assay. Empty MSs did not inhibit COX catalytic activity, excluding any inhibitory effect derived from the polymeric matrix (Table S3). Among all samples, the only preparation that showed an inhibitory activity, albeit low, was MS/K-1, which corresponds to the preparation with the K highest content and slowest release. The maximum percentage of inhibition was observed after 24 h, when the amount of released K was expected to be highest (Figure 10b). Comparable inhibition was recorded after 1 h and 5 h.

If the aim of the formulation was to achieve controlled drug release of K, these preliminary findings are consistent with such release profiles. Specifically, K at a concentration of 7.0 × 10^−9^ M and 3.5 × 10^−9^ M produced approximately 15% and 1% COX-1 inhibition, respectively (Figure 10c). Based on this concentration–response relationship, the K concentration released after 24 h was estimated to be approximately 4.2 × 10^−9^ M in DMSO, compared to 2.5 × 10^−9^ M calculated in the PBS release test. However, this comparison should be done carefully. K concentrations were esteemed from the UV–Vis release study in PBS while COX inhibition assays were performed to indirectly esteem the inhibitory response of samples prepared in DMSO. Thus, data cannot be directly compared as equivalent quantitative measurements. The two assays were conducted in different media and relied on distinct analytical principles. Differences in K solubility, molecular environment, extraction efficiency, and assay sensitivity may therefore contribute to the discrepancy between the two estimated concentrations. In particular, DMSO may facilitate the diffusion of K from the MS matrix, whereas the release assay in PBS reflects only the fraction released under aqueous physiological-like conditions. Although the estimated K concentration released after 24 h is unlikely to produce a pharmacologically relevant anti-inflammatory effect, the ability of the released drug to inhibit COX-1, even to a limited extent (approximately 4%), demonstrates that K retained its biological activity following encapsulation and release. Therefore, the COX inhibition assay should be regarded as an indirect functional confirmation of drug release and retained activity rather than as evidence of therapeutic efficacy.

These results demonstrate that biologically active K was released from the MS. However, as known from literature, the amount released is not sufficient to achieve a therapeutically relevant anti-inflammatory effect. Further optimization of the formulation will therefore be required to increase drug loading and/or modulate the release profile to achieve pharmacologically relevant local drug concentrations.

### 3.11. Evaluation of MS/K Adhesion to a Wound Model by Dry Spray

**Figure 11:**
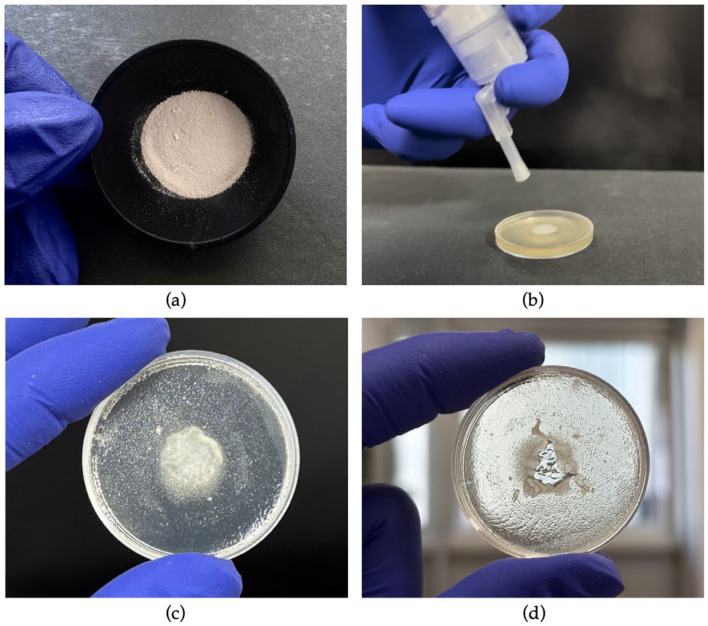
Lyophilized MS/K powder before application (**a**); deposition of MS/K on a simulated wound model by means of a non-pressurized spray bottle for dry powders (**b**); MS/K retained the SWF and coalesced into a film 10 min after deposition (**c**); inversion test showing the absence of visible macroscopic detachment of the formed layer after sample flipping under static testing conditions, followed by scratching of the MS/K layer to highlight its film-like structure (**d**).

The MS/K adhesion to a wound model was preliminary in vitro evaluated. The lyophilized MS/K were loaded into a non-pressurized spray bottle for dry powders without any added excipients and deposited on a wound model filled with SWF (Figure 11b). Upon contact with the moist wound bed, MS/K rapidly hydrated and swelled by absorbing the SWF, forming a uniform, adhesive, and adherent film within 10 min. The macroscopic appearance of the lyophilized MS/K powder (Figure 11a) and its behavior during (Figure 11b) and after (Figure 11c) deposition onto the simulated wound model was reported in Figure 11. After deposition, MS/K fully adsorbed the SWF within 10 min and remained associated with the gelatin/SWF substrate during the observation period (up to 1 h), with no visible macroscopic detachment after multiple inversion tests. During the inversion tests, sample flipping did not result in material loss or detachment. Scratching of the MS/K layer was then performed to highlight its film-like structure (Figure 11d). These findings provide preliminary qualitative evidence that the dry MS/K formulation can hydrate and remain associated with a simplified moist substrate under static in vitro conditions.

## 4. Discussion and Conclusions

This study proposed a method to produce gelatin MSs crosslinked with TA for the encapsulation and controlled release of K in the context of difficult-to-heal wounds, where prolonged inflammation compromises tissue regeneration. The proposed single-step W/O emulsification method allowed to produce round-like MSs with yields that varied from 19% to 42%. Morphological and dimensional characterization via electron microscopy and laser diffraction demonstrated the presence of MSs of about 10–40 µm in a dry state (20–80 µm in full swelled state). Process optimization revealed that microsphere morphology and size distribution are predominantly governed by emulsification parameters (rpm and time) rather than by the TA:PHC molar ratio. Moderate shear (3000 rpm) MSs promoted the formation of the most homogeneous MSs, while the highest investigated speeds (10,000 rpm) introduced polydispersity, likely due to shear-induced instability in droplet formation. In parallel, reduced emulsification times (5 min) led to irregular particles, confirming that sufficient mixing time (at least 10 min) is essential for stable droplet generation. Additionally, crosslinking conditions (time and TA:PHC molar ratio) were shown to strongly modulate MS network stability. Shorter crosslinking times (0.5 h) resulted in higher aggregation, weaker morphology, and lower synthesis yields. FT-IR analysis showed small shifts in the amide I and II bands, indicating PHC–TA interactions. These may involve hydrogen bonding as well as hydrophobic, π–π, van der Waals, and possibly covalent interactions. However, ATR-FTIR cannot distinguish or quantify the contribution of each mechanism [78,80,81,82,119,120]. Crosslinking degree showed high values (>90%) in most formulations, with longer crosslinking times producing significantly denser networks. The swelling degree closely mirrored crosslinking trends. MSs crosslinked for 4 h showed the highest water absorption capacity (~1791–2100%), while shorter times (<2 h) led to poor swelling due to incomplete network formation. Even if these data seemed to be opposite to standard polymer science principles, it must be taken into account that partial degradation of MSs occurred before 24 h. Thus, the apparent lower swelling degree of poorly crosslinked MSs must be interpreted as a phenomenon due to MS partial degradation and to a consequent reduced material recovery. Degradation profiles validated the critical role of crosslinking time. Only formulations crosslinked for more than 2 h significantly retained their structure after 6 h, whereas 0.5 h samples degraded rapidly. All these findings provided complementary evidence of the successful formation of the tannic acid-crosslinked gelatin network. Although no single technique can directly quantify all molecular interactions involved in the crosslinking process, the combination of ATR-FTIR analysis, crosslinking degree determination, swelling behavior, and degradation studies allowed a reliable indirect assessment of the extent of network formation. In particular, the progressive increase in crosslinking degree, accompanied by the corresponding changes in swelling capacity and degradation resistance, clearly demonstrates the strong influence of crosslinking time on the structural organization and stability of the microspheres. Nevertheless, authors acknowledge that thermal analysis on developed MS by differential scanning calorimetry (DSC) would provide important additional insights on the structural stabilization of gelatin induced by tannic acid. The thermal behavior of the crosslinked network as well as the influence of the degree of crosslinking on the thermal resistance and the denaturation temperature of gelatin will thus be object of further future in-depth evaluations.

After investigation of key processing parameters of MS properties, K encapsulation attempts were performed. Comparative characterization tests with empty MSs were performed. The successful encapsulation of K was evidenced by FT-IR analysis by the presence of ketonic and ester vibrations in MS/K spectra, without evidence of drug degradation. Crosslinking degree showed high values (>90%) confirming that K loading did not reduce CD%. The loading of MSs with K did not compromise structural integrity nor modify MS surface roughness, as visualized in SEM images. No significant shifts in yield were observed, as well as in MS diameter distribution both in dry and hydrated conditions. MS/K also retained high swelling ratios, confirming that drug presence does not hinder hydration. Drug EE% and in vitro release tests were then performed. EE% varied from less than 15% to nearly 39%, showing a strong dependence from crosslinking time. The most stable MS/K-1 formulation retained significantly more drug, likely due to reduced porosity and higher network cohesion forces. As expected, in vitro release studies showed that K diffusion is tightly controlled by matrix density. MS/K-1 provided sustained release (62% at 24 h), while MS/K-3 released the entire load within 3 h. Kinetic modeling supported a predominantly diffusion-driven, near-Fickian release for MS/K-1 and a more anomalous contribution for MS/K-2, while MS/K-3 showed burst release and could not be reliably classified by Korsmeyer–Peppas analysis. However, it should be acknowledged that the Korsmeyer–Peppas analysis should be considered preliminary, as the model is valid only for the initial stage of release (typically below 60% cumulative release), and the limited number of experimental data points within this range reduces the robustness of the fitted parameters. Therefore, the calculated release exponent (n) values should be interpreted with caution. Nevertheless, the observed differences in n suggest that MS/K-1 is predominantly governed by Fickian diffusion, whereas the higher n value obtained for MS/K-2 may indicate the contribution of additional mechanisms, such as polymer matrix relaxation and swelling, resulting in anomalous transport. Although the good fitting of release kinetics with applied mathematical models, these findings should be regarded as preliminary qualitative indications of the prevailing release mechanisms rather than definitive mechanistic evidence of real kinetics. COX inhibition assays confirmed that K released from MS/K-1 retained pharmacological activity under the assay conditions. However, these data were interpreted only as complementary functional evidence and not as a direct quantitative measure of K release, because the COX assay was performed in DMSO whereas release testing was performed in PBS. Under the tested conditions, TA and unloaded MS did not show detectable COX inhibition in the assay, confirming that the observed activity was attributable to K.

Finally, a preliminary MS/K adhesion test was performed on a simulated wound model. In the simplified gelatin/SWF wound model, dry MS/K rapidly hydrated and coalesced into a film-like layer that remained associated with the substrate under static conditions. Although positive, these findings provide only preliminary evidence supporting the feasibility of the proposed formulation for topical administration. The performed assay was intended solely as a qualitative proof-of-concept and does not provide quantitative information regarding bioadhesive performance. Future studies should therefore include quantitative bioadhesion assays together with more physiologically relevant ex vivo and in vivo wound models to comprehensively evaluate the retention and therapeutic performance of the developed MS under clinically relevant conditions.

The cytocompatibility test demonstrated the non-cytotoxicity of the gelatin-based microspheres. However, it should be acknowledged that although positive this evaluation represents only a preliminary assessment of their biological safety. Cell metabolic activity alone cannot provide a comprehensive understanding of the biological response elicited by the material. In depth in vitro and in vivo investigations are mandatory to assess the biocompatibility profile of the proposed delivery system. Further tests like live/dead staining, adhesion analyses, and long-term proliferation studies are some examples. In addition, given the intended biomedical application, it would be valuable to investigate the interactions of the microspheres with the immune system, including their potential to modulate phagocytic activity and macrophage uptake. Likewise, studies addressing the interaction of the microspheres with human biological fluids would provide important insights into how the material surface may influence biological recognition, degradation behavior, and therapeutic performance under physiological conditions. In this regard, the determination of MSs’ zeta potential will give important complementary information. These investigations were beyond the scope of the present work but represent important directions for future research aimed at supporting the clinical translation of this delivery platform.

In conclusion, this study provides a solid proof-of-concept for the development of TA-crosslinked gelatin MSs as a biocompatible and tuneable platform for sustained K delivery in wound management. All findings highlight the versatility of the proposed system and support its potential application in wound settings, contributing to the development of localized anti-inflammatory therapies with improved safety and efficacy profiles. This system could potentially contribute to the development of safer and more effective topical therapeutic strategies.

## Figures and Tables

**Table 1:** Investigated processing parameters of MS and MS/K.

Cod.	Homogenization Speed (rpm)	Homogenization Time (min)	TA: PHC Molar Ratio	Crosslinking Time (h)
MS-1	1000	5	1:2	4
MS-2	1000	10	1:2	4
MS-3	1000	15	1:2	4
MS-4	3000	5	1:2	4
MS-5	3000	10	1:2	4
MS-6	3000	15	1:2	4
MS-7	10,000	5	1:2	4
MS-8	10,000	10	1:2	4
MS-9	10,000	15	1:2	4
MS-10	3000	15	1:2	2
MS-11	3000	15	1:2	0.5
MS-12	3000	15	1:4	4
MS-13	3000	15	1:4	2
MS-14	3000	15	1:4	0.5
MS/K-1	3000	15	1:2	4
MS/K-2	3000	15	1:2	2
MS/K-3	3000	15	1:2	0.5

**Table 2:** Effects of the variations of synthesis conditions on MSs and MS/K yield and CD%. Reported values represent the mean ± SD (*n* = 3).

Cod.	Yield (%)	CD%
MS-1	30 ± 3	95.7 ± 1.7
MS-2	34 ± 6	97.0 ± 1.5
MS-3	36 ± 4	97.1 ± 1.8
MS-4	26 ± 5	96.4 ± 1.9
MS-5	34 ± 4	96.5 ± 1.2
MS-6	36 ± 3	95.6 ± 2.3
MS-7	30 ± 2	96.1 ± 1.7
MS-8	42 ± 4	94.8 ± 1.9
MS-9	45 ± 4	96.2 ± 1.9
MS-10	23 ± 2	90.5 ± 1.8
MS-11	28 ± 4	86.0 ± 2.2
MS-12	36 ± 2	96.1 ± 0.9
MS-13	25 ± 3	96.0 ± 0.5
MS-14	20 ± 4	86.8 ± 3.2
MS/K-1	31 ± 2	95.8 ± 1.2
MS/K-2	27 ± 4	93.9 ± 2.7
MS/K-3	24 ± 3	90.9 ± 0.4

**Table 3:** Kinetic-model fitting of K release from MS/K formulations.

Cod.	Zero-Order (R^2^)	First-Order (R^2^)	Higuchi (R^2^)	Korsmeyer–Peppas (n)	Korsmeyer–Peppas (R^2^)
MS/K-1	0.018	0.451	0.884	0.440	0.945
MS/K-2	0.175	0.950	0.786	0.675	0.979
MS/K-3	−0.932	0.916	0.240	-	-

## Data Availability

The original contributions presented in this study are included in the article/Supplementary Materials. Further inquiries can be directed to the corresponding author.

## Supplementary Materials

The following supporting information can be downloaded at: https://www.mdpi.com/article/10.3390/polym18151807/s1, Figure S1: Representative SEM micrographs of samples obtained after the emulsion process performed without the addition of a crosslinking agent. No stable PHC-based MS were formed or recovered, demonstrating that crosslinking is essential for the formation and structural stabilization of MS; Figure S2: Cell viability percentage estimated through MTT assay performed with NIH/3T3 cells after 24 h incubation of free K at different concentrations; Table S1: Size distribution of dry MS in terms of average diameter (Dm), followed by D10, D50, D90 and span index esteemed from SEM analysis; Table S2: Size distribution of wet MS in terms of average diameter (Dm), followed by D10, D50, D90, and span index esteemed from SEM analysis; Table S3: Tannic acid was tested at four concentrations corresponding to those used in the drug-release kinetics study (0.011–0.099 M). Since no concentration-dependent effects were observed, only the COX-1 and COX-2 inhibition values obtained at the highest concentration tested (0.099 M) are reported. Empty MSs were tested at the concentration used in the formulation (1 mg/mL). No inhibition of either COX-1 or COX-2 was observed after 1, 5, or 24 h of incubation. Since the results were identical at all time points, only the inhibition values obtained after 24 h are reported.

## Abbreviations

The following abbreviations are used in this manuscript:

DDS	Drug Delivery Systems
NSAID	Non-Steroidal Anti-Inflammatory Drugs
COX	Cyclooxygenase
K	Ketoprofen
MS	Microspheres
TA	Tannic Acid
DMSO	Dimethyl sulfoxide
FTIR	Fourier-Transform Infrared Spectroscopy
PHC	Partially Hydrolyzed Type I Collagen
SEM	Scanning Electron Microscope
CD%	Degree of Crosslinking
PBS	Phosphate-Buffered Saline
SD%	Degree of Swelling
BCA	Bicinchoninic Acid
DR%	Degradation Resistance
EE%	Encapsulation Efficiency
R%	Percentage of drug release
SWF	Simulated Wound Fluid
SD	Standard Deviation
MTT	3-(4,5-dimethylthiazol-2-yl)-2,5-diphenyltetrazolium bromide
NIH/3T3	Embryonic Mouse Fibroblasts
HaCaT	Human Keratinocytes
