# Supplementary material for: Gelatin-Based Microspheres for Sustained Ketoprofen Delivery in Difficult-to-Heal Wounds
Source: Polymers (Basel). 2026 Jul 23;18(15):1807. doi: 10.3390/polym18151807 (PMC13468595; doi:10.3390/polym18151807)
Supplement: Supplementary file 1 [file polymers-18-01807-s001.zip › polymers-4414040-supplementary.pdf]

polymers

MDPI

Supplementary Materials

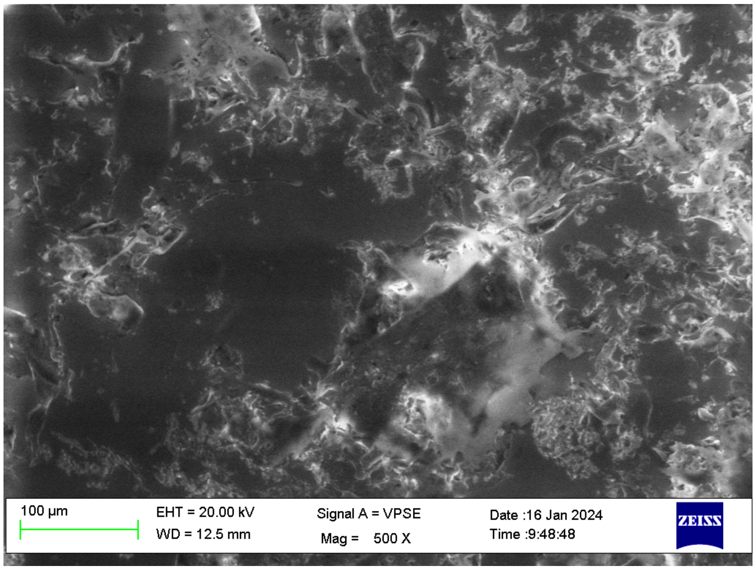

Figure S1. Representative SEM micrographs of samples obtained after the emulsion process performed without the addition of a crosslinking agent. No stable PHC-based MS were formed or recovered, demonstrating that crosslinking is essential for the formation and structural stabilization of MS.

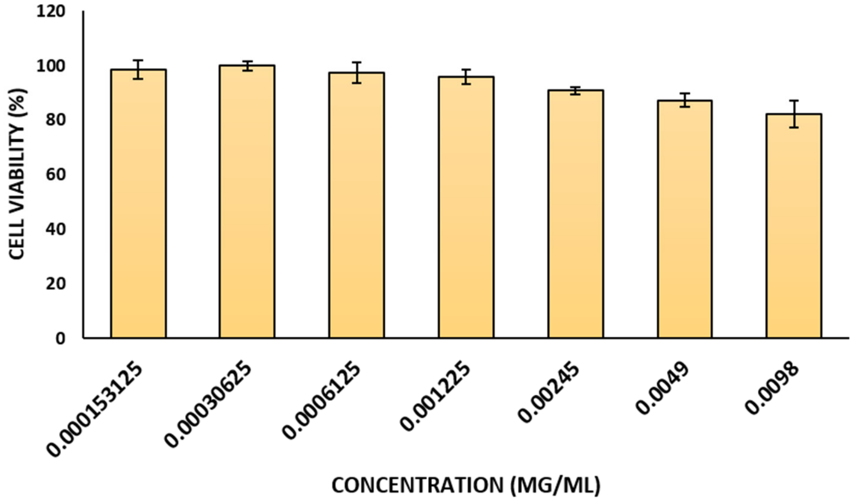

Figure S2. Cell viability percentage estimated through MTT assay performed with NIH/3T3 cells after 24 hours incubation of free K at different concentrations.

 https://doi.org/10.3390/polym18151807

Table S1. Size distribution of dry MS in terms of average diameter (Dm), followed by D10, D50, D90, Dm and span index esteemed from SEM analysis. Reported values represent the mean ± SD (n = 3).

Cod.	D10	D50	D90	Dm	Span index
MS-1	10.1 ± 0.7	18.9 ± 2.1	31.6 ± 3.6	20.2 ± 3.9	1.3 ± 0.5
MS-2	6.1 ± 0.5	9.6 ± 1.3	22.7 ± 2.2	11.3 ± 2.5	1.7 ± 0.3
MS-3	4.7 ± 0.4	7.3 ± 0.8	17.7 ± 2.7	9.1 ± 2.2	1.8 ± 0.2
MS-4	10.3 ± 0.2	17.1 ± 1.1	33.9 ± 1.6	19.6 ± 3.4	1.4 ± 0.4
MS-5	6.3 ± 0.5	12.7 ± 0.9	24.9 ± 4.4	14.6 ± 2.3	1.5 ± 0.2
MS-6	5.9 ± 0.3	11.9 ± 0.8	24.6 ± 3.2	14.1 ± 1.7	1.6 ± 0.2
MS-7	8.2 ± 0.2	14.3 ± 1.3	30.5 ± 5.1	18.6 ± 4.2	1.6 ± 0.1
MS-8	6.8 ± 0.6	10.3 ± 2.4	24.1 ± 1.9	12.9 ± 2.5	1.7 ± 0.2
MS-9	4.4 ± 1.1	7.8 ± 2.8	15.6 ± 3.3	9.1 ± 2.1	1.5 ± 0.1
MS-10	7.5 ± 0.5	13.9 ± 0.1	24.4 ± 1.4	15.1 ± 0.3	1.2 ± 0.1
MS-11	6.9 ± 0.6	11.6 ± 0.5	19.0 ± 0.3	12.2 ± 0.5	1.0 ± 0.1
MS-12	5.9 ± 0.1	10.0 ± 0.8	15.3 ± 0.9	10.5 ± 0.9	0.93 ± 0.01
MS-13	6.7 ± 0.9	10.7 ± 1.7	16.6 ± 1.6	11.8 ± 1.5	0.9 ± 0.1
MS-14	11.3 ± 1.7	18.4 ± 3.7	31.4 ± 3.5	20.3 ± 3.1	1.1 ± 0.1
MS/K-1	9.0 ± 0.2	11.8 ± 2.3	19.9 ± 2.2	12.8 ± 2.7	0.9 ± 0.2
MS/K-2	11.1 ± 0.1	15.4 ± 4.2	20.9 ± 3.2	15.8 ± 2.1	0.6 ± 0.2
MS/K-3	9.7 ± 0.3	15.6 ± 3.5	21.7 ± 3.6	16.2 ± 1.1	0.78 ± 0.1

Table S2. Size distribution of wet MS in terms of average diameter (Dm), followed by D10, D50, D90, Dm and span index esteemed from laser diffraction analysis. Reported values represent the mean ± SD (n = 3).

Cod.	D10	D50	D90	Dm	Span index
MS-1	10.3 ± 1.7	32.7 ± 3.2	67.1 ± 4.7	36.2 ± 2.9	1.7 ± 0.1
MS-2	16.3 ± 2.8	44.3 ± 2.8	87.3 ± 3.1	48.6 ± 2.7	1.6 ± 0.1
MS-3	14.5 ± 0.1	36.2 ± 1.1	73.2 ± 3.7	40.4 ± 1.6	1.6 ± 0.1
MS-4	3.6 ± 0.9	29.9 ± 5.3	77.1 ± 31.5	37.7 ± 12.7	2.4 ± 0.6
MS-5	13.5 ± 0.7	35.0 ± 1.8	68.9 ± 2.9	38.3 ± 1.7	1.6 ± 0.1
MS-6	*10.5 ± 4.6*	29.9 ± 4.1	59.9 ± 5.3	33.0 ± 4.3	1.7 ± 0.2
MS-7	9.0 ± 3.0	32.1 ± 7.8	65.8 ± 17.0	35.5 ± 8.9	1.77 ± 0.03
MS-8	14.0 ± 1.4	38.5 ± 3.5	76.3 ± 6.3	42.3 ± 3.7	1.63 ± 0.03
MS-9	*12.3 ± 0.6*	30.8 ± 2.5	102.7 ± 74.2	45.0 ± 20.6	2.9 ± 2.2
MS-10	*12.5 ± 3.2*	33.9 ± 3.1	60.4 ± 5.3	35.1 ± 5.2	1.4 ± 0.2
MS-11	11.5 ± 5.2	32.4 ± 2.1	65.3 ± 4.2	38.4 ± 3.6	1.7 ± 0.2
MS-12	13.1 ± 1.5	34.6 ± 2.9	71.8 ± 5.4	39.7 ± 3.1	1.7 ± 0.1
MS-13	9.8 ± 2.2	31.5 ± 3.7	66.9 ± 6.1	36.0 ± 4.0	1.8 ± 0.2
MS-14	15.2 ± 1.9	40.1 ± 3.0	79.4 ± 4.8	44.8 ± 3.4	1.6 ± 0.4
MS/K-1	14.8 ± 1.1	38.9 ± 2.4	74.6 ± 3.9	42.1 ± 2.7	1.5 ± 0.1
MS/K-2	12.9 ± 2.6	35.2 ± 3.6	69.8 ± 5.7	39.4 ± 4.2	1.6 ± 0.1
MS/K-3	10.9 ± 3.8	31.0 ± 4.9	58.7 ± 6.8	34.8 ± 5.1	1.6 ± 0.2

Table S3. [a]Tannic acid was tested at four concentrations corresponding to those used in the drug-release kinetics study (0.011–0.099 M). Since no concentration-dependent effects were observed, only the COX-1 and COX-2 inhibition values obtained at the highest concentration tested (0.099 M) are reported. [b]Empty MS were tested at the concentration used in the formulation (1 mg/mL). No

https://doi.org/10.3390/polym18151807

inhibition of either COX-1 or COX-2 was observed after 1, 5, or 24 h of incubation. Since the results were identical at all time points, only the inhibition values obtained after 24 h are reported.

	(% inhibition)	
**Sample**	**COX-1**	**COX-2**
TA[a]	0	0
eMS[b]	0	0

Disclaimer/Publisher's Note: The statements, opinions and data contained in all publications are solely those of the individual author(s) and contributor(s) and not of MDPI and/or the editor(s). MDPI and/or the editor(s) disclaim responsibility for any injury to people or property resulting from any ideas, methods, instructions or products referred to in the content.

https://doi.org/10.3390/polym18151807
